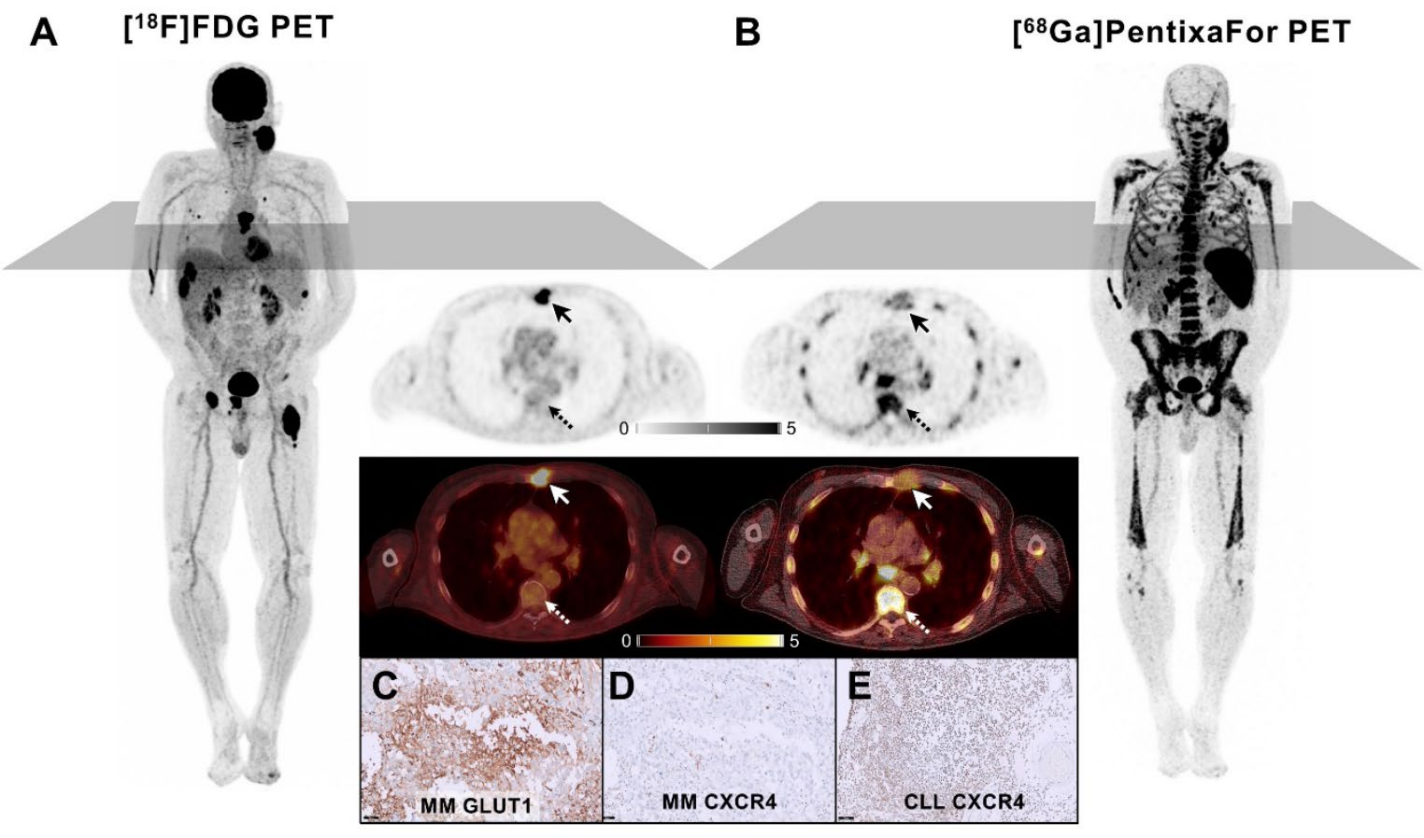# Disclosing tumor biology by means of molecular imaging in a patient with malignant melanoma and chronic lymphocytic leukemia

**DOI:** 10.1007/s00259-024-06834-3

**Published:** 2024-07-15

**Authors:** Alexander Gäble, Johanna S. Enke, Martin J. Hügle, Przemyslaw Grochowski, Martin Trepel, Alexander Dierks, Christian H. Pfob, Ralph A. Bundschuh, Constantin Lapa, Malte Kircher

**Affiliations:** 1 Nuclear Medicine, Faculty of Medicine, University of Augsburg, Augsburg, Germany; 2 Pathology, Faculty of Medicine, University of Augsburg, Augsburg, Germany; 3 Hematology and Oncology, Faculty of Medicine, University of Augsburg, Augsburg, Germany; 4 Bavarian Cancer Research Center (BZKF), Erlangen, Germany

## Figure

C-X-C motif chemokine receptor 4 (CXCR4) plays a major role in tumor growth and the process of metastasis and is thus a highly attractive target in oncology [1]. Non-invasive chemokine receptor imaging using positron emission tomography (PET) has demonstrated promising results, especially in hematologic malignancies including multiple myeloma or lymphoma [2]. Tumor detection in solid cancers is more heterogeneous (as compared to [^18^F]-fluorodeoxyglucose ([^18^F]FDG)) with many entities showing only low to moderate in vivo CXCR4 expression [3, 4].

A 73-year-old male with a history of Binet Stage B chronic lymphatic leukemia (CLL), and lentigo maligna melanoma (UICC IA) presented for re-staging with a rapidly enlarging cervical mass as well as new liver lesions (detected by previous ultrasound). [^18^F]FDG-PET/computed tomography (CT) displayed high uptake in the cervical mass and liver lesions. In addition, pulmonary and osteolytic bone lesions with intense [^18^F]FDG accumulation could be detected (A). In contrast, CT showed various additional enlarged lymph nodes and splenomegaly with only minimal [^18^F]FDG uptake (A). Thus, CXCR-directed imaging was added. Contrary to [^18^F]FDG, [^68^Ga]Ga-PentixaFor displayed intense tracer uptake -consistent with CLL- in the enlarged lymph nodes, bone marrow and spleen, and minimal uptake in the aforementioned [^18^F]FDG-avid sites, suggestive of melanoma metastases (B). Immunohistochemical staining confirmed these findings showing high GLUT1 (C) and low CXCR4 expression (D) in an osteolytic melanoma lesion (arrows; SUV_max_ 14.0 vs. 2.2) as opposed to intense CXCR4 expression (E) in a site with osseous CLL infiltration (dotted arrows; SUV_max_ 2.6 vs. 9.1). Our case highlights the ability of molecular imaging to non-invasively phenotype disease and visualize tumor biology.